# Carrier Frequency of Autosomal Recessive Diseases in a Population Attending a Human Fertility Institute in Colombia

**DOI:** 10.5935/1518-0557.20240102

**Published:** 2025

**Authors:** Germán David Ospina Idárraga, Iván Darío Montes Suárez, Lina Maria Caicedo Muriel, Katherine Gisell Hernández Osorio, Diana Milena Diaz Corredor, Paola Andrea Montealegre

**Affiliations:** 1 InSer Institute of Human Fertility, Bogotá, Colombia; 2 El Country Clinical Gynecology Department. GISE Menopause and Clinical Gynecological Endocrinology of the Country. Bogota Colombia

**Keywords:** recessive diseases, mutation, assisted reproduction treatment, carrier screening, variants frequently

## Abstract

**Objective:**

To determine the carrier frequency of X-linked and autosomal recessive diseases in patients attending a human fertility institute in Colombia.

**Methods:**

This retrospective observational study included patients and gamete donors attending a Human Fertility Institute in Colombia between January 2017 and June 2023. Sociodemographic data and results of Next Generation Sequencing laboratory panels for screening of recessive disease-causing mutations were collected and analyzed.

**Results:**

Data from 746 samples were analyzed; 599 (80.3%) were Colombian origin individuals and 147 (19.7%) were foreigners. At least one mutation was detected in 526 (70.5%) individuals. Of note, 893 pathogenic genetic variants were identified.

The genetic variants most frequently observed in all the individuals studied were associated with the following diseases (carrier frequency): alpha thalassemia (10.5%), alpha-1 antitrypsin deficiency (10%), congenital adrenal hyperplasia due to 21-hydroxylase deficiency (9.4%), cystic fibrosis (7.3%), spinal muscular atrophy type 1 (5.6%) and Stargardt disease type 1 (5.0%). The most frequent genetic variant observed in the subgroup of Colombian origin individuals was associated with alpha-1 antitrypsin deficiency (11.3%).

**Conclusions:**

Information on the frequency of recessive diseases in Colombia is limited. This pioneering carrier genetic screening identified a high percentage of carriers for at least one recessive autosomal or X-linked in the population evaluated. Screening for recessive mutations could lead to an evolution in family planning programs and a decrease in the number of patients affected by recessive disorders. Furthermore, it could become a routine test not only in cases of assisted reproduction but also in cases of natural gestation.

## INTRODUCTION

Carrier screening (CS) was introduced in the 1970s to identify gene mutations related with the transmission of autosomal recessive or X-linked diseases (Kraft *et al*., 2019; Payne *et al*., 2021). Initially, CS tests were limited to specific ethnic groups with an increased frequency of autosomal recessive disease, for example, Jewish communities in which Tay-Sachs disease is prevalent (Harper *et al*., 2018). Over time, CS has been extended to the general population, and in 2010, expanded carrier genetic tests (CGTs) based on next-generation sequencing (NGS) technology were introduced to detect a larger number of genetic mutations (Srinivasan *et al*., 2010).

Recessive diseases are conditions caused by mutations of genes located on autosomal or sexual chromosomes. An individual manifests the disease by inheriting two mutated alleles, one from each parent. When an individual harbors one normal allele and one mutated allele, he/she is known as a carrier and does not have the disease. If both parents carry the mutated gene, the probability that their offspring will inherit two abnormal copies and thus develop the disease is 25%. Inbreeding is the leading risk factor for recessive diseases, as both parents are more likely to be carriers of the same genetic mutation (Gross & Gheorghe, 2020).

Currently, CGTs play an essential role in assisted reproductive technology (ART) procedures for infertility treatment. Individuals who choose ART methods using their own or donated gametes, as well as fertility centers, consider the evaluation of recessive mutations relevant because of the possibility of transmitting genetic diseases to offspring (Serrano-Serrano, 2012). CGT results are unique to each individual and serve to establish the risk of their offspring developing an autosomal recessive disease (Gregg *et al*., 2021). CGT helps individuals to decide autonomously about their reproductive choices.

The CGT is carried out using next-generation sequencing (NGS) based laboratory panels. This technology facilitates the universal screening of many mutations in coding regions of genes associated with recessive diseases. Thanks to improved technology, high demand of genetic testing in the market, and massification of NGS-based tests, the cost of CGT has progressively decreased over time (Reguera, 2021).

Recessive mutations can be detected in peripheral blood samples. Deoxyribonucleic acid (DNA) is extracted and purified from blood cells. Then, targeted exons are identified, amplified to construct libraries, and massively sequenced using a standardized platform. Subsequent bioinformatics analysis makes it possible to detect the presence of specific mutations (Reguera, 2021). Finally, the clinical relevance of the results is built on gene databases that include genetic variants classified as pathogenic (with strong evidence of association with the disease) or likely pathogenic (probably responsible for causing disease, although scientific evidence for such an association is insufficient (Rubio *et al*., 2020).

Carrier genetic testing panels help to identify single nucleotide changes and insertions and deletions of less than 20 base pairs in exons. However, there are technical limitations that make it challenging to identify mutations such as large deletions, duplications, inversions, ploidy changes, mosaicism, epigenetic alterations, germline mutations, chromosomal abnormalities, mitochondrial DNA mutations, non-coding region variants, pseudogenes, and genes with partial sequence coverage (Reguera, 2021).

Mutations identified by the genetic screening panels must have been previously published by the Human Gene Mutation DATABASE (HGMD^®^). Scientific societies such as the American College of Medical Genetics and Genomics (ACMG), the European Society of Human Reproduction and Embryology (ESHRE), and the Spanish Fertility Society (SEF) publish their own criteria for defining the diseases to be included in the carrier genetic screening (Martin *et al*., 2015).

The prevalence of autosomal recessive diseases varies according to geographic location and ethnicity of the populations. For example, sickle cell anemia is highly prevalent due to 21-hydroxylase deficiency (21-OHD) has a carrier rate of 1:60 in the general population (Huidobro Fernández *et al*., 2012) The incidence of cystic fibrosis (CF) is approximately 1:2500 in individuals of European descent (Scotet *et al*., 2020). Notably, the prevalence of Tay-Sachs disease is high (1:3600) in Ashkenazi Jewish population compared to other populations (1:320 000) (Xiao & Lauschke, 2021).

In Colombia, the frequency of genetic diseases ranges between 37.3 and 52.8 per 1000 inhabitants (Bernal & Suárez, 1996). Studies reveal that the prevalence of some recessive diseases follows a characteristic pattern depending on the ethnicity of the populations. For example, recessive diseases are more frequent in rural areas of Boyacá, Santander, and Antioquia departments due to their high prevalence of consanguinity (De Castro & Restrepo, 2015).

Information on the prevalence of genetic diseases in Colombia is scarce. The prevalence of CF has been estimated to be higher than 1:12 000 individuals; however, this indicator could be underestimated due to the delay in diagnosis, the high number of undiagnosed cases, and the early mortality caused by the disease (De Castro & Restrepo, 2015) Variable data on hemoglobinopathies have been published. Romero-Sánchez et al. retrospectively studied (2009 - 2012) a group of patients from different Colombian cities with suspected hemoglobinopathy. They reported a frequency of hemoglobinopathy of 34.5%, with thalassemia as the most frequent quantitative hemoglobinopathy (Romero-Sánchez *et al*., 2015). In another retrospective study of 2224 individuals, Vargas-Hernandez et al. observed that the prevalence of thalassemia was 14.3% (Vargas-Hernández *et al*., 2023). Another investigation conducted in Cali evaluated a cohort of 152 patients (0-18 years aged), and reported a frequency of hemoglobinopathies of 42.7%, with sickle cell trait being the most frequent variant (14.5%), followed by sickle cell disease (11.8%) (Aguirre *et al*., 2020). A recent study in a cohort of 1107 patients with chronic obstructive pulmonary disease revealed that 13.01% of them had alpha-1 antitrypsin deficiency (Alí-Munive *et al*., 2023).

The miscegenation in Colombia -originated from migration of different populations during the last five centuries-has resulted in a high incidence of mutations. These mutations are frequent in some geographical areas of the country due to genetic drift and the impact of colonization. However, information on the prevalence of other autosomal recessive or X-linked diseases, such as spinal muscular atrophy (SMA), congenital adrenal hyperplasia, Tay-Sachs disease, phenylketonuria, or Wilson disease, is scarce in Colombia.

This study aimed to determine the carrier frequency of autosomal recessive or X-linked diseases in individuals attending a human fertility institute in Colombia. For this purpose, data from patients and gamete donors who underwent CGT were collected and analyzed.

## MATERIAL AND METHODS

### Study design

This retrospective observational study included 746 individuals attending the inSer Human Fertility Institute clinics in Bogotá, Medellín, Pereira, and Cartagena between January 2017 and June 2023. Data from individuals who underwent CGT for autosomal recessive or X-linked diseases were collected and analyzed.

The study was approved by the medical and research ethics committee of inSer Human Fertility Institute clinics, ensuring the compliance of the research process to ethical standards and those defined in the 1975 Helsinki declaration, revised in 2013. All patients filled out and signed an institutional and reference laboratory informed consent form, accepting the procedures, risks and possible complications.

### Study population

Patients and gamete donors who underwent CGT for autosomal recessive or X-linked diseases as part of the institutional protocol before ART procedures.

### Carrier Genetic Testing

Peripheral blood samples were collected and sent to Igenomix and Sistemas Genómicos/Synlab for CGT. The following CGT panels were used:

**CGT PLUS**^®^ (Igenomix): Expanded panel that analyzes 470 genes in males and 536 (66 X-linked) in females. It determines the presence of more than 30 000 genetic variants and more than 500 diseases.

**Preconception *Focus GeneProfile^®^*** (Sistemas Genómicos): This panel identifies more than 8 000 variants in 299 genes, responsible for 332 autosomal recessive diseases and 31 X-linked diseases.

**Preconception universal *GeneProfile***^**®**^ (Sistemas Genómicos): This exome-targeted test analyzes variants of 298 genes, corresponding to 331 autosomal recessive diseases and 31 X-linked diseases.

### Statistical analysis

Results for all individuals were retrieved from Igenomix and Sistemas Genómicos. A database was constructed with the following variables: Identification, age, sex, type of individual (patients and gamete donors), place of origin, date of examination, the laboratory that carried out the screening, type of genetic screening panel, genetic variants detected, number of pathogenic genetic variants, mutated genes, and associated diseases.

The “place of origin” variable was initially categorized as continents and subcontinents. However, as many patients came from the Americas, the categories “North America”, “Central America”, “South America”, and “the Antilles” were added.

A descriptive analysis of the qualitative variables was done using absolute and relative frequencies. According to data distribution, quantitative variables were described as mean and standard deviation (SD) or median and interquartile range (IQR).

## RESULTS

The study included 746 individuals aged 18 to 76 years; 376 (50.4%) were men, 370 (49.5%) were women; 495 (66.3%) were patients, and 251 (33.6%) were gamete donors; 599 (80.3%) were Colombians, and 147 (19.7%) were foreigners (Table 1).

**Table 1:** Distribution of the study population according to place of origin.

Continent or subcontinent	n (%)
North America	34 (4.5)
Central America	4 (0.6)
South America	615 (82.4)
Asia	10 (1.3)
Australia	4 (0.6)
Europe	63 (8.4)
Antilles	16 (2.2)

Blood samples from gamete donors and patients for CGT were sent to Sistemas Genómicos and Igenomix laboratories. Sistemas Genómicos analyzed 641 (85.9%) samples, and Igenomix analyzed 105 (14.1%) samples.

Of the individuals studied, 526 (70.5%) were positive for at least one pathogenic genetic variant. Pathogenic genetic variants per individual varied between 1-6 (Mean=1.70; 95%CI=1.62-1.77) (Table 2). A total of 893 pathogenic genetic variants were identified (Table 3).

**Table 2:** Distribution of pathogenic genetic variants detected by the CGT panels.

CGT panel	Number of pathogenic genetic variants detected	
	Yes	No
CGT plus^®^	87	18
Preconception *Focus GeneProfile*^®^	398	186
Preconception universal GeneProfile^®^	41	16
Total	**526**	**220**

**Table 3:** Number of pathogenic genetic variants per individual.

Number of variants	n (%)
1	273 (51.9)
2	161 (30.6)
3	75 (14.2)
4-6	17 (3.3)
**Total**	**526 (100)**

The most frequently observed diseases in the analyzed population were: alpha thalasemia (α-thalassemia) (10.5%), alpha-1 antitrypsin deficiency (10%), congenital adrenal hyperplasia due to 21-OHD (9.4%), CF (7.3%), SMA type 1 (5.6%) and Stargardt disease type 1 (5.0%) (Table 4). In the Colombian subgroup, the most frequently observed diseases were: alpha-1 antitrypsin deficiency (11.3%), congenital adrenal hyperplasia due to 21-OHD (10.2%), α-thalassemia (10%), CF (6.9%), SMA type 1 (6.1%) and Stargardt disease type 1 (5.1%) (Table 5).

**Table 4:** Gene frequency in the study population.

Gene	Disease	n	%
*HBA2*	Alpha thalassemia	86	9.6
*HBA*	Alpha thalassemia	5	0.6
*HBA1/HBA2*	Alpha thalassemia	2	0.2
*HBA1*	Alpha thalassemia	1	0.1
*SERPINA1*	Alpha-1 antitrypsin deficiency	89	10.0
*CYP21A2*	Congenital adrenal hyperplasia due to 21-hydroxylase deficiency	84	9.4
*CFTR*	Cystic fibrosis	65	7.3
*SMN1*	Spinal muscular atrophy type 1	50	5.6
*ABCA4*	Stargardt's disease type 1; cone-rod dystrophy type 3	45	5.0
*GJB2*	Autosomal recessive non-syndromic sensorineural deafness, type DFNB	18	2.0
*G6PD*	Glucose-6-phosphate dehydrogenase deficiency	17	1.9
*ATP7B*	Wilson's disease	16	1.8
*CBS*	Homocystinuria due to cystathionine beta-synthase deficiency	14	1.6
*CFTR*	Bilateral congenital absence of the vas deferens	13	1.5
*OCA2*	Oculocutaneous albinism type 2	13	1.5
*PMM2*	Congenital disorder of glycosylation type 1a	13	1.5
*GAA*	Glycogen storage disease due to acid maltase deficiency (Pompe disease)	12	1.3
*ACADM*	Medium-chain acyl-CoA dehydrogenase deficiency	11	1.2
*SBDS*	Shwachman-Diamond Syndrome	11	1.2
*TYR*	Oculocutaneous albinism type 1A	11	1.2
*SLC26A2*	Atelosteogenesis type 2	8	0.9
*BTD*	Biotinidase deficiency	7	0.8
*GALC*	Krabbe disease	7	0.8
*HBB*	Sickle cell anemia	7	0.8
*ADA*	Severe combined immunodeficiency due to adenosine deaminase (ADA) deficiency	6	0.7
*BCHE*	Butyrylcholinesterase deficiency	6	0.7
*CLCN1*	Autosomal recessive myotonia congenita	6	0.7
*COL7A1*	Dystrophic epidermolysis bullosa (EAD) Hallopeau-Siemens (HS) and non-HS type; puriginous EAD; pretibial EDA	6	0.7
*GJB2*	Autosomal recessive hearing loss type 1A; Digenic hearing loss GJB2/GJB6	6	0.7
*HBB*	Beta thalassemia	6	0.7
*HEXA*	Tay/Sachs disease	6	0.7
*MEFV*	Mediterranean fever	6	0.7
*SPG7*	Autosomal recessive spastic paraplegia type 7	6	0.7
*TMPRSS3*	Autosomal recessive non-syndromic sensorineural deafness, type DFNB10	6	0.7
*ACADSB*	2-methylbutyryl-CoA dehydrogenase deficiency	5	0.6
*DHCR7*	Smith-Lemli-Opitz syndrome	5	0.6
*FIG4*	Charcot-Marie-Tooth disease type 4J	5	0.6
*GBA*	Gaucher disease	5	0.6
*GDAP1*	Charcot-Marie-Tooth disease type 4A	5	0.6
*GNPTAB*	Mucolipidosis type 2 alpha/beta; Mucolipidosis type 3 alpha/beta	5	0.6
*SMPD1*	Niemann-Pick disease type A; Niemann-Pick disease type B	5	0.6
*ACADS*	Short-chain acyl-CoA dehydrogenase deficiency	4	0.4
*CNGB3*	Acromatopsia 3	4	0.4
*FAH*	Tyrosinemia type 1	4	0.4
*FMR1*	Fragile X syndrome	4	0.4
*GNRHR*	Hypogonadotropic hypogonadism type 7	4	0.4
*HBB*	HBB-related hemoglobinopathies	4	0.4
*PAH*	Phenylketonuria	4	0.4
*POLR1C*	Treacher Collins syndrome	4	0.4
*ALDOB*	Hereditary fructose intolerance	3	0.3
*AR*	Partial androgen insensitivity syndrome	3	0.3
*ARSA*	Metachromatic leukodystrophy	3	0.3
*CAPN3*	Autosomal recessive limb-girdle muscular dystrophy type 2A	3	0.3
*COL4A3*	Autosomal recessive Alport syndrome	3	0.3
*CYP27A1*	Cerebrotendinous xanthomatosis	3	0.3
*DCLRE1C*	Omenn syndrome; Severe combined immunodeficiency Athabaskan type	3	0.3
*GALNS*	Mucopolysacchariosis type 4A	3	0.3
*OTOF*	Autosomal recessive nonsyndromic sensorineural deafness, type DFNB9	3	0.3
*PKHD1*	Polycystic kidney disease type 4	3	0.3
*RPGRIP1L*	Joubert syndrome type 7; Meckel syndrome type 5; COACH Syndrome	3	0.3
*SLC22A5*	Systemic primary carnitine deficiency	3	0.3
*SPG11*	Juvenile amyotrophic lateral sclerosis, type 5	3	0.3
*UGT1A1*	Crigler-Najjar syndrome type 2	3	0.3
*USH2A*	Retinitis pigmentosa 39	3	0.3
*USH2A*	Usher syndrome type 2A	3	0.3
*AGL*	Glycogen storage disease type 3	2	0.2
*AIPL1*	Leber congenital amaurosis 4	2	0.2
*ARSB*	Mucopolysaccharidosis type 6 (Maroteaux-Lamy syndrome)	2	0.2
*ASS1*	Citrullinemia type 1	2	0.2
*CEP290*	Meckel syndrome type 4; Joubert syndrome type 5; Leber congenital amaurosis type 10	2	0.2
*CHST6*	Macular corneal dystrophy 1	2	0.2
*CNGB1*	Retinitis pigmentosa 45	2	0.2
*CPT2*	Carnitine palmitoyl palmitoyltransferase deficiency type 2, neonatal lethal form; Carnitine palmitoyltransferase deficiency type 2, infantile form	2	0.2
*CRB1*	Autosomal recessive retinitis pigmentosa 12	2	0.2
*CYP1B1*	Primary congenital glaucoma type 3A	2	0.2
*DYSF*	Miyoshi muscular dystrophy type 1; autosomal recessive limb-girdle muscular dystrophy type 2 (LGMD2)	2	0.2
*GALE*	Galactose epimerase deficiency	2	0.2
*GALT*	Galactosemia	2	0.2
*GCDH*	Glutaric acidemia type 1	2	0.2
*HGSNAT*	Mucopolysacchariosis type 3, Sanfilippo Syndrome type C	2	0.2
*LOXHD1*	Autosomal recessive deafness type 77	2	0.2
*MYO15A*	Autosomal recessive deafness type 3	2	0.2
*MYO7A*	Usher syndrome type 1B; autosomal recessive deafness type 2	2	0.2
*NR2E3*	Enhanced Cone-S syndrome (Goldmann-Favre); Retinitis pigmentosa type 37	2	0.2
*SLC26A4*	Autosomal recessive deafness type 4; Pendred Syndrome	2	0.2
*SLC6A19*	Hartnup disease	2	0.2
*SUMF1*	Multiple sulfatase deficiency	2	0.2
*TRDN*	Catecholaminergic polymorphic ventricular tachycardia 5	2	0.2
*ABCA3*	Pulmonary surfactant metabolism disfunction type 3	1	0.1
*ACADVL*	Very long chain acyl-CoA dehydrogenase deficiency	1	0.1
*ACSF3*	Combined malonic and methylmalonic acidemia	1	0.1
*AHI1*	Joubert syndrome 3	1	0.1
*AHCY*	Hypermethioninemia with S-adenosyl homocysteine (AdoHcy) hydrolase deficiency	1	0.1
*AGXT*	Primary hyperoxaluria type 1	1	0.1
*ALG3*	Congenital disorder of glycosylation type Id	1	0.1
*ALG12*	Congenital disorder of glycosylation type Ig	1	0.1
*ALG1*	Congenital disorder of glycosylation type 1K	1	0.1
*ASL*	Argininosuccinic aciduria	1	0.1
*ATM*	Ataxia telangiectasia	1	0.1
*CERKL*	Retinitis pigmentosa 26	1	0.1
*CLN3*	Neuronal ceroid lipofuscinosis type 3	1	0.1
*CTNS*	Nephropathic cystinosis	1	0.1
*DBT*	Maple syrup urine disease 2	1	0.1
*DMD*	Duchenne muscular dystrophy	1	0.1
*DUOX2*	Familial thyroid dyshormonogenesis type 6	1	0.1
*DOK7*	Fetal akinesia deformation sequence type 3; congenital myasthenic syndrome type 10	1	0.1
*DNAH5*	Primary ciliary dyskinesia type 3	1	0.1
*ETFDH*	Glutaric acidemia 2C	1	0.1
*F11*	Autosomal recessive factor XI deficiency	1	0.1
*EIF2B5*	Leukoencephalopathy with vanishing white matter	1	0.1
*EYS*	Retinitis pigmentosa type 25	1	0.1
*FH*	Fumaric aciduria	1	0.1
*FANCG*	Fanconi anemia complementation group G	1	0.1
*GAMT*	Cerebral creatine deficiency syndrome type 2	1	0.1
*GBE1*	Glycogen storage disease type 4	1	0.1
*GBBS10*	Bardet Bieldl syndrome type 1	1	0.1
*GFM1*	Hepatoencephalopathy due to combined oxidative phosphorylation deficiency type 1	1	0.1
*GLA*	Fabry disease	1	0.1
*HADHA*	Deficiency of 3-hydroxyacyl-CoA dehydrogenase of long-chain fatty acids	1	0.1
*GRHPR*	Primary hyperoxaluria type 2	1	0.1
*HDD*	Sickle cell anemia	1	0.1
*HOGA1*	Primary hyperoxaluria type 3	1	0.1
*HEXB*	Sandhoff disease - infantile, juvenile and adult forms	1	0.1
*IVD*	Isovaleric acidemia	1	0.1
*HPD*	Tyrosinemia type 3	1	0.1
*LHFPL5*	Autosomal recessive non-syndromic sensorineural deafness, type DFNB67	1	0.1
*IDUA*	Hurler syndrome	1	0.1
*MPI*	Congenital disorder of glycosylation type 1b	1	0.1
*MMACHC*	Methylmalonic acidemia with homocystinuria type cblC	1	0.1
*MVK*	Mevalonic aciduria	1	0.1
*MPDU1*	Congenital disorder of glycosylation 1F	1	0.1
*MTHFR*	Homocystinuria due to MTHFR deficiency	1	0.1
*NPHP1*	Nephronophthisis	1	0.1
*NLRP7*	Recurrent hydatidiform mole type 1	1	0.1
*NPC2*	Niemann-Pick disease type C2	1	0.1
*NPC1*	Niemann-Pick disease type C1	1	0.1
*PCDH15*	Usher syndrome type 1F	1	0.1
*PEX12*	Peroxisome biogenesis disorder type 3A (Zellweger spectrum)	1	0.1
*PCCA*	Propionic acidemia	1	0.1
*POLG*	POLG-realated disorders	1	0.1
*RDH12*	Leber congenital amaurosis 13	1	0.1
*PROP1*	Panhypopituitarism	1	0.1
*RAG2*	Combined immunodeficiency with skin granulomatosis	1	0.1
*RARS2*	Pontocerebellar hypoplasia type 6	1	0.1
*POMT1*	Congenital muscular dystrophy-dystroglycanopathy type 1A (Walker/ Warburg syndrome); type 1B; Type 1C (autosomal recessive limb-girdle muscular dystrophy type 11 (LGMD R11)	1	0.1
*SAG*	Oguchi disease	1	0.1
*SCO2*	Fatal infantile cardioencephalomyopathy due to cytochrome c oxidase 1 deficiency.	1	0.1
*SGSH*	Mucopolysaccharidosis type 3A	1	0.1
*SH3TC2*	Charcot-Marie-Tooth disease type 4C	1	0.1
*SGCA*	Autosomal recessive limb-girdle muscular dystrophy type 3 (LGMD R3)	1	0.1
*SLC12A3*	Gitelman syndrome	1	0.1
*ST3GAL5*	Salt and pepper developmental regression syndrome (Amish infantile epileptic syndrome)	1	0.1
*TPP1*	Juvenile neuronal ceroid lipofuscinosis	1	0.1
*TREX1*	Aicardi-Goutieres syndrome type 1	1	0.1
*UGT1A1*	Crigler-Najjar syndrome type 1	1	0.1

**Table 5:** Gene frequency in Colombian subgroup.

Gene	Disease	n	%
*SERPINA1*	Alpha-1 antitrypsin deficiency	80	11.3
*CYP21A2*	Congenital adrenal hyperplasia due to 21-hydroxylase deficiency	72	10.2
*HBA2*	Alpha thalassemia	67	9.5
*HBA1/HBA 2*	Alpha thalassemia	2	0.3
*HBA1*	Alpha thalassemia	1	0.1
*HBA*	Alpha thalassemia	1	0.1
*CFTR*	Cystic fibrosis	49	6.9
*SMN1*	Spinal muscular atrophy type 1	43	6.1
*ABCA4*	Stargardt's disease type 1; cone-rod dystrophy type 3	36	5.1
*GJB2*	Autosomal recessive non-syndromic sensorineural deafness, type DFNB	15	2.1
*ATP7B*	Wilson's disease	14	2.0
*CBS*	Homocystinuria due to cystathionine beta-synthase deficiency	13	1.8
*CFTR*	Bilateral congenital absence of the vas deferens	13	1.8
*G6PD*	Glucose-6-phosphate dehydrogenase deficiency	13	1.8
*ACADM*	Medium-chain acyl-CoA dehydrogenase deficiency	11	1.6
*OCA2*	Oculocutaneous albinism type 1A	10	1.4
*PMM2*	Congenital disorder of glycosylation type 1a	10	1.4
*TYR*	Oculocutaneous albinism type 1A	9	1.3
*SBDS*	Shwachman-Diamond Syndrome	8	1.1
*SLC26A2*	Atelosteogenesis type 2	8	1.1
*GAA*	Glycogen storage disease due to acid maltase deficiency (Pompe disease)	7	1.0
*CLCN1*	Autosomal recessive myotonia congenita	6	0.8
*COL7A1*	Dystrophic epidermolysis bullosa (EAD) Hallopeau-Siemens (HS) and non-HS type; puriginous EAD; pretibial EDA	6	0.8
*HEXA*	Tay/Sachs disease	6	0.8
*MEFV*	Mediterranean fever	6	0.8
*TMPRSS3*	Autosomal recessive non-syndromic sensorineural deafness, type DFNB10	6	0.8
*ADA*	Severe combined immunodeficiency due to adenosine deaminase (ADA) deficiency	5	0.7
*BCHE*	Butyrylcholinesterase deficiency	5	0.7
*BTD*	Biotinidase deficiency	5	0.7
*FIG4*	Charcot-Marie-Tooth disease type 4J	5	0.7
*GDAP1*	Charcot-Marie-Tooth disease type 4A	5	0.7
*HBB*	Sickle cell anemia	5	0.7
*ACADS*	Short-chain acyl-CoA dehydrogenase deficiency	4	0.6
*FAH*	Tyrosinemia type 1	4	0.6
*FMR1*	Fragile X syndrome	4	0.6
*GALC*	Krabbe disease	4	0.6
*GNPTAB*	Mucolipidosis type 2 alpha/beta; Mucolipidosis type 3 alpha/beta	4	0.6
*GNRHR*	Hypogonadotropic hypogonadism type 7	4	0.6
*HBB*	Beta thalassemia	4	0.6
*POLR1C*	Treacher-Collins syndrome	4	0.6
*SPG7*	Autosomal recessive spastic paraplegia type 7	4	0.6
*ACADSB*	2-methylbutyryl-CoA dehydrogenase deficiency	3	0.4
*ALDOB*	Hereditary fructose intolerance	3	0.4
*CNGB3*	Acromatopsia 3	3	0.4
*COL4A3*	Autosomal recessive Alport syndrome	3	0.4
*DCLRE1C*	Omenn syndrome; Severe combined immunodeficiency Athabaskan type	3	0.4
*GALNS*	Mucopolysacchariosis type 4A	3	0.4
*GJB2*	Autosomal recessive hearing loss type 1A; Digenic hearing loss GJB2/GJB6	3	0.4
*OTOF*	Autosomal recessive nonsyndromic sensorineural deafness, type DFNB9	3	0.4
*PAH*	Phenylketonuria	3	0.4
*RPGRIP1L*	Joubert syndrome type 7; Meckel syndrome type 5; COACH Syndrome	3	0.4
*SMPD1*	Niemann-Pick disease type A; Niemann-Pick disease type B	3	0.4
*UGT1A1*	Crigler-Najjar syndrome type 2	3	0.4
*AGL*	Glycogen storage disease type 3	2	0.3
*AIPL1*	Leber congenital amaurosis 4	2	0.3
*AR*	Partial androgen insensitivity syndrome	2	0.3
*ARSB*	Mucopolysaccharidosis type 6 (Maroteaux-Lamy syndrome)	2	0.3
*CAPN3*	Autosomal recessive limb-girdle muscular dystrophy type 2A	2	0.3
*CHST6*	Macular corneal dystrophy 1	2	0.3
*CNGB1*	Retinitis pigmentosa 39	2	0.3
*CYP27A1*	Cerebrotendinous xanthomatosis	2	0.3
*DHCR7*	Smith-Lemli-Opitz syndrome	2	0.3
*DYSF*	Miyoshi muscular dystrophy type 1; autosomal recessive limb-girdle muscular dystrophy type 2 (LGMD2)	2	0.3
*GALE*	Galactose epimerase deficiency	2	0.3
*GBA*	Gaucher disease	2	0.3
*HGSNAT*	Mucopolysacchariosis type 3, Sanfilippo Syndrome type C	2	0.3
*MYO15A*	Autosomal recessive deafness type 3	2	0.3
*NR2E3*	Enhanced Cone-S syndrome (Goldmann-Favre); Retinitis pigmentosa type 37	2	0.3
*PKHD1*	Polycystic kidney disease type 4	2	0.3
*SLC22A5*	Systemic primary carnitine deficiency	2	0.3
*SPG11*	Juvenile amyotrophic lateral sclerosis, type 5	2	0.3
*USH2A*	Retinitis pigmentosa 39	2	0.3
*USH2A*	Usher syndrome type 2A	2	0.3
*ACADVL*	Very long chain acyl-CoA dehydrogenase deficiency	1	0.1
*ACSF3*	Combined malonic and methylmalonic acidemia	1	0.1
*AHI1*	Joubert syndrome 3	1	0.1
*ALG3*	Congenital disorder of glycosylation type Id	1	0.1
*ALG12*	Congenital disorder of glycosylation type Ig	1	0.1
*ARSA*	Metachromatic leukodystrophy	1	0.1
*ATM*	Ataxia telangiectasia	1	0.1
*ASS1*	Citrullinemia type 1	1	0.1
*CERKL*	Retinitis pigmentosa 26	1	0.1
*CEP290*	Meckel syndrome type 4; Joubert syndrome type 5; Leber congenital amaurosis type 10	1	0.1
*CPT2*	Carnitine palmitoyl palmitoyltransferase deficiency type 2, neonatal lethal form; Carnitine palmitoyltransferase deficiency type 2, infantile form	1	0.1
*CYP1B1*	Primary congenital glaucoma type 3A	1	0.1
*DMD*	Duchenne muscular dystrophy	1	0.1
*DOK7*	Fetal akinesia deformation sequence type 3; congenital myasthenic syndrome type 10	1	0.1
*DNAH5*	Primary ciliary dyskinesia type 3	1	0.1
*FH*	Fumaric aciduria	1	0.1
*FANCG*	Fanconi anemia complementation group G	1	0.1
*GALT*	Galactosemia	1	0.1
*GAMT*	Cerebral creatine deficiency syndrome type 2	1	0.1
*GCDH*	Glutaric acidemia type 1	1	0.1
*GBBS10*	Bardet Bieldl syndrome type 1	1	0.1
*GFM1*	Hepatoencephalopathy due to combined oxidative phosphorylation deficiency type 1	1	0.1
*GLA*	Fabry disease	1	0.1
*HADHA*	Deficiency of 3-hydroxyacyl-CoA dehydrogenase of long-chain fatty acids	1	0.1
*GRHPR*	Primary hyperoxaluria type 2	1	0.1
*HBB*	HBB-related hemoglobinopathies	1	0.1
*HDD*	Sickle cell anemia	1	0.1
*IVD*	Isovaleric acidemia	1	0.1
*IDUA*	Hurler syndrome	1	0.1
*LOXHD1*	Autosomal recessive deafness type 77	1	0.1
*HOGA1*	Primary hyperoxaluria type 3	1	0.1
*MMACHC*	Methylmalonic acidemia with homocystinuria type cblC	1	0.1
*MVK*	Mevalonic aciduria	1	0.1
*MPDU1*	Congenital disorder of glycosylation 1F	1	0.1
*MTHFR*	Homocystinuria due to MTHFR deficiency	1	0.1
*NLRP7*	Recurrent hydatidiform mole type 1	1	0.1
*NPC1*	Niemann-Pick disease type C1	1	0.1
*PCDH15*	Usher syndrome type 1F	1	0.1
*PEX12*	Peroxisome biogenesis disorder type 3A (Zellweger spectrum)	1	0.1
*PCCA*	Propionic acidemia	1	0.1
*POLG*	POLG-realated disorders	1	0.1
*PROP1*	Panhypopituitarism	1	0.1
*POMT1*	Congenital muscular dystrophy-dystroglycanopathy type 1A (Walker/ Warburg syndrome); type 1B; Type 1C (autosomal recessive limb-girdle muscular dystrophy type 11 (LGMD R11)	1	0.1
*SAG*	Oguchi disease	1	0.1
*SCO2*	Fatal infantile cardioencephalomyopathy due to cytochrome c oxidase 1 deficiency.	1	0.1
*SGSH*	Mucopolysaccharidosis type 3A	1	0.1
*SH3TC2*	Charcot-Marie-Tooth disease type 4C	1	0.1
*SLC12A3*	Gitelman syndrome	1	0.1
*SLC26A4*	Autosomal recessive deafness type 4; Pendred Syndrome	1	0.1
*SUMF1*	Multiple sulfatase deficiency	1	0.1
*TRDN*	Catecholaminergic polymorphic ventricular tachycardia 5	1	0.1
*TREX1*	Aicardi-Goutieres syndrome type 1	1	0.1
*UGT1A1*	Crigler-Najjar syndrome type 1	1	0.1

## DISCUSSION

The CGT has been done for more than 30 years to detect recessive diseases in the general population (Gregg, 2018). Advances in genomics have made it possible to evolve from identifying point mutations to universal, rapid, and efficient screening of genetic variants. Today, NGS-based CGTs makes it possible to detect different disease-causing mutations in a single test and has even led to the discovery of new clinically relevant genetic variants (Reguera, 2021).

In 2016, Anna Abulí et al. studied a cohort of 1301 individuals from a reproductive program in Barcelona. The cohort comprised 635 (48.8%) male couples undergoing ART with donated eggs, 483 (37.1%) egg donor candidates, 105 (8.1%) female couples undergoing ART with donated sperm, and 39 (6.0%) heterosexual couples who attended for preconception examination or treated by ART with own gametes. All individuals underwent CGT for 200 genes associated with 368 (277 autosomal recessive, 37 X-linked, and 54 autosomal dominant) disorders. The results showed that 733/1331 (56.3%) individuals carried at least one pathogenic or likely pathogenic mutation. In addition, 1.7% of the egg donors were carriers of X-linked diseases. The five most frequent autosomal recessive diseases (gene associated, number, and [percentage] of carriers) were GJB2-related DFNB1 nonsyndromic hearing loss (*GJB2*, 69 [5.3%]); CF (*CFTR*, 45, [3.5%]); α-thalassemia, (*HBA1/HBA2*, 40, [3.1%]); phenylketonuria (*PAH*, 39 [3.0%]); and spinal muscular atrophy (*SMN1*, 37 [2.8%]) (Abulí *et al*., 2016).

In 2020, Xi Yanping et al. conducted a prospective cohort study in a Chinese fertility clinic to evaluate the CGT results of 2923 patients undergoing ART treatment. The CGT panel analyzed 201 genes associated with 135 recessive (autosomal or X-linked) diseases. The results showed that 46.73% of individuals carried at least one pathogenic or likely pathogenic mutation. They found that 2836 patients with no family history carried genetic variants associated with the following diseases (number and [frequency] of carriers): citrin deficiency (111 [3.91%]), GJB2-related DFNB1 nonsyndromic hearing loss (106 [3.74%]), Krabbes disease (80 [2.82%]), Usher syndrome type 2A (76 [2.68%]), α-thalassemia (66 [2.33%]), and Wilson disease (66 [2.33%]) (Xi *et al*., 2020).

In 2021, Ngoc Hieu Tran et al. analyzed CGT results for recessive diseases in a cohort of 985 Vietnamese individuals by clinical exome sequencing of 4503 genes. They identified 118 recessive diseases associated with 164 pathogenic or likely pathogenic variants. The most prevalent recessive disorders (gene associated; carrier frequency) were: autosomal recessive deafness (*GJB2*; 17.2%), Cohen syndrome (*VPS13B*; 4.5%), beta-thalassemia (*HBB*; 4.3%), citrin deficiency (*SLC25A13*; 3.2%), cataract 13 with adult I phenotype (*GCNT2*; 3.74%), Joubert syndrome (T*MEM67*; 2.6%), and phenylketonuria (*PHA*; 2.5%) (Tran *et al*., 2021).

In contrast to the studies above, the carrier frequency of at least one mutation in the population analyzed in the present study was 70.5%. This figure is higher than those reported in a Chinese group (46.73%) (Xi *et al*., 2020) and in Barcelona (56.3%) (Abulí *et al*., 2016). This difference can be attributed to the miscegenation characteristic of the population here evaluated and to the inbreeding occurring in some regions of Colombia.

It is striking that some of the highest recessive disease frequencies observed in the present study were α-thalassemia (10.5%), alpha-1 antitrypsin deficiency (10%), congenital adrenal hyperplasia due to 21-OHD (9.4%), cystic fibrosis (7.3%), spinal muscular atrophy type 1 (5.6%) and Stargardt disease type 1 (5.0%). Interestingly, some of these diseases were also the most frequent in Abulí’s study in Barcelona: cystic fibrosis (3.5%), αthalassemia (3.1%), and spinal muscular atrophy type 1 (2.8%). This result could not be attributed to chance but to the Spanish colonization of Colombian lands more than five centuries ago.

Published information on recessive diseases in Colombia is limited. Most of it refers to specific diseases and is found in non-indexed journals. Data on the frequency of disease carriers derived from expanded genetic screening is lacking. The present study is one of the first to describe the Carrier Frequency of Autosomal Recessive Diseases in a presumably healthy population. The main pathogenic or likely pathogenic mutations detected in Colombians and foreigners were associated with the following diseases, in order of frequency: α-thalassemia, alpha-1 antitrypsin deficiency, congenital adrenal hyperplasia due to 21-OHD, CF, SMA type 1 and Stargardt disease type 1. In turn, the main mutations identified in the Colombian population were related to the following diseases in order of frequency: alpha-1 antitrypsin deficiency, congenital adrenal hyperplasia due to 21-OHD, α-thalassemia, CF, SMA type 1 and Stargardt disease type 1. Descriptions of these disorders are briefly described below.

### Alpha 1 antitrypsin deficiency

This disease is associated with chronic obstructive pulmonary disease (COPD), mainly of the emphysematous type, liver disease (cirrhosis), and, less frequently, panniculitis and vasculitis. The gene encoding alpha-1-antitrypsin has several alleles, which are transmitted to offspring by simple Mendelian inheritance with autosomal codominant behavior. Most individuals (85 - 90%) inherit the normal alleles, designated M. The deficient alleles, designated S and Z, have a variable prevalence according to geographical location. In Europe, the disease is more prevalent (1:1500-2000) in the northwestern coastal regions, gradually decreasing eastward (1:10 000 - 1:90 000) and disappearing closer to Asia. In America, the prevalence of the disease is highest among northern Caucasian populations (1:5000 - 6000 individuals) and decreases five times in the south of the continent. In Argentina, depending on the combination of alleles, the prevalence of alpha 1 antitrypsin deficiency varies between 1:2400 and 1:26 000 individuals (Menga *et al*., 2014). In Colombia, a study of 2023 reported that genetic mutations (M/S - M/Z) were 13% of a group of patients with COPD (Alí-Munive *et al*., 2023).

### Congenital adrenal hyperplasia due to alpha 21-OHD

Congenital adrenal hyperplasia comprises autosomal recessive disorders of adrenal steroidogenesis. Between 90 - 99% of cases result from mutations in the *CYP21A2* gene (Chr 6p21.3), which encodes the enzyme 21-hydroxylase. The disease is characterized by deficient production of sex steroids but normal gonadal development. Virilization of the female external genitalia is observed, with variable degrees of clitoral enlargement and labial fusion (Claahsen-van der Grinten *et al*., 2022). More than 230 *CYP21A2* pathogenic variants have been identified (Kocova *et al*., 2022).

The global estimated prevalence of 21-hydroxylase deficiency (21-OHD) is 1:60 but may be as high as 1:3 in communities with a smaller gene pool. The highest prevalence rates are observed in China and India, and the lowest in Japan and New Zealand (Kocova *et al*., 2022). In the United States, 21-OHD has an incidence of approximately 1:15 000 in whites, but it is more prevalent among Native Americans and Yupik Eskimos (Momodu *et al*., 2023). In 2023, Navarro-Zambrana and Sheets reported that according to the neonatal screening performed in 31 countries (58 studies between 1969 and 2017), the global incidence of 21-OHD was 1:9498; highest in the Eastern Mediterranean and Southeast Asia (> 15:100 000) and lowest in the Western Pacific countries of Asia (< 5:100 000). No significant differences were observed in Hispanic/Latino and white groups but a higher incidence was observed in individuals of African descent. In Latin America, Argentina had the highest incidence (1:8937) (Navarro-Zambrana & Sheets, 2023). Information on prevalence, frequency, and incidence of congenital adrenal hyperplasia due to alpha 21-OHD in Colombia is limited.

### Alpha thalassemia

The α-thalassemia is an autosomal recessive hemoglobinopathy that affects 5% of the global population. The disease is most prevalent in China, India, Africa, Southeast Asia, and the Middle East. However, migratory patterns have contributed to the worldwide distribution of the disease (Horvei *et al*., 2021). In California (UEA), 1 in 10 000 children is born with clinically significant α-thalassemia (Horvei *et al*., 2021). The frequency of α-thalassemia carriers varies by geographic region; in tropical and subtropical areas, it can be as high as 80% - 90% (Farashi & Harteveld, 2018).

The α-thalassemia results from mutations in the alpha globin genes (1 to 4 genes) (Chr 16p) (Horvei *et al*., 2021). The disease is caused by a deficiency in the synthesis of the α-globin chains of hemoglobin, resulting in decreased hemoglobin concentration and anemia. The severity of the disease depends on the number of lacking genes. It can range from the asymptomatic form, through α-thalassemia minor, hemoglobin H disease, to α-thalassemia major, called hemoglobin Bart hydrops fetalis (Hb Bart) syndrome (Farashi & Harteveld, 2018). There are no studies on the frequency of α-thalassemia in Colombia.

### Cystic fibrosis

Cystic fibrosis is an autosomal recessive monogenic disease of worldwide distribution. The incidence, carrier rate, and prevalent mutation vary according to the population studied, with Caucasians being the most affected (López-Valdez *et al*., 2021). Cystic fibrosis originates as a consequence of pathogenic changes in the *CFTR* gene (Chr7q.31), which encodes the protein known as cystic fibrosis transmembrane conductance regulator (CFTR) (López-Valdez *et al*., 2021; Salcedo *et al*., 2012). Dysfunction of this protein alters the ion transport on the apical surface of epithelial cells, mainly affecting the lungs, pancreas, liver, intestine, and testes.

Since 1938, when CF was described, 2114 *CFTR* mutations have been identified (http://www.genet.sickkids.on.ca/cftr/StatisticsPage.html; accessed on August 16, 2023), the most frequent being the delta F508. This mutation is present in 70% to 80% of Americans affected by the disease (Acuña *et al*., 2004), and although to a lesser degree, it is also the most common in Mexico, Venezuela, and Colombia. According to a collaborative study of these three countries, the frequency of the delta F508 mutation ranged from 29.63% to 47.7% (Pérez *et al*., 2007). Another study in Colombia with a larger number of patients reported that the delta F508 mutation had a frequency of 28%, with wide regional variations. Although such mutation was the most frequent, it does not exceed 40% (Restrepo *et al*., 2000).

The only actual figure for CF in Colombia comes from a neonatal screening performed in Bogotá in 2011. This genetic screening for CF -with the immunoreactive trypsinogen (IRT) test, followed by molecular studies- showed that CF has an incidence of 1:8297 newborns in our country (Amado González, 2011).

### Spinal muscular atrophy type 1

It is one of the most frequent monogenic neurodegenerative diseases. Its global incidence is estimated to be between 1:6000 and 1:10 000 live births (Lunn & Wang, 2008; Ogino *et al*., 2002; Schorling *et al*., 2020; Verhaart *et al*., 2017). Approximately 95% of cases of SMA are caused by deletions or point mutations in the *SMN1* (Surviving Motor Neuron 1) gene (Chr5q11.2-5q13.3) (5q-SMA). The remaining cases are due to mutations in other genes (non-5q-SMA) (Schorling *et al*., 2020). *SMN1* mutations cause the absence of functional SMN protein, leading to the degeneracy of alpha motor neurons in the spinal cord and, thus, weakness, atrophy, and eventually, muscle paralysis (Mercuri *et al*., 2018).

Different phenotypes of SMA have been described. They are related to the age of symptom onset and the maximal motor ability achieved. These phenotypes depend on the number of copies of the *SMN2* gene (support gene). SMA type 1, which begins before six months of age, is the most severe form with more neurological compromise. Without treatment and ventilatory support, it is the leading cause of death due to genetic neurodegenerative disease in early childhood, with a life expectancy of less than two years (Farrar *et al*., 2013; Zerres & Schöneborn, 1995). SMA type 2, with onset between 6 and 18 months of age and milder symptoms, manifests in children able to sit up on their own but unable to walk. In SMA type 3, symptoms develop with varying degrees of weakness, joint contracture, scoliosis, and loss of ambulation beginning in infancy or adolescence. SMA type 4 manifests in adulthood and has a similar development to SMA type 3, although it progresses more slowly (Mercuri *et al*., 2018).

Identification of a couple’s carrier status, together with genetic counseling, allows for adequate pregnancy planning. *In vitro* fertilization and preimplantation genetic studies increase the probability of having a healthy child (Keinath *et al*., 2021). Few studies on SMA have been published in Colombia; precise statistics on its incidence or prevalence are needed (Cardona *et al*., 2022).

### Stargardt disease

Hereditary retinal dystrophy (HRD) comprises a group of diseases that cause degeneracy of the photoreceptors, both cones and rods. These specialized cells play a crucial role in biological processes such as phototransduction and the visual cycle.

According to different studies, the global prevalence for this disease varies from 1:8000 to 1:10 000 (46). In Colombia, 2 cases (0.03%) were reported in the 2022 orphan/rare disease report (SIVIGILA) (SIVIGILA, 2022).

Stargardt disease is the most common form of macular dystrophy. It is an autosomal recessive disorder caused by mutations in the *ABCA4* (*STGD1*) gene (Del Pozo-Valero *et al*., 2020). Affected individuals manifest central and bilateral centrifugal vision loss and macular atrophy due to subretinal deposition of lipofuscin-like substances (Huang *et al*., 2022). The age of onset is an indirect marker of prognosis: the earlier the onset, the more severe the disease course (Tsang & Sharma, 2018).

Clinical diagnosis is difficult to make when patients do not consult early. In advanced stages, the phenotype of retinal dystrophies is often very similar, and the diagnosis may be inadequate. However, new molecular diagnostic tools can help confirm a Stargardt disease diagnosis (Acevedo, 2021).

Studies on indicators of disease, disability, and death due to hereditary disorders are limited in Colombia (Grisales-Romero *et al*., 2018); however, the effects of these disorders are potentially fatal or debilitating in the long term. Although inherited diseases may have a low incidence, they collectively affect 6% of the global population and represent a significant family, social, and economic burden (del Pilar Ramírez Rey, 2013). Preconception screening by CGT and prenatal counseling are valuable tools to reduce the risk of transmitting recessive diseases to offspring, as well as their social and economic cost (Verma & Puri, 2015).

In recent years, ART procedures have evolved to impact family planning significantly. ART procedures have conventionally been considered an alternative for treating infertility. However, over time, they have become valuable tools to help determine how and when to achieve a successful pregnancy. Vitrification of oocytes, sperm, and embryos, for example, allows individuals to use such biological material, at least in theory, whenever they deem it relevant throughout their lives. Although still a controversial topic, new generations of patients and healthcare providers are more aware of the existence of ART procedures and consider them useful for planning their families by medically assisted means. ART procedures are undoubtedly related to advances in reproductive genetics, in which CGT plays a key role. Future generations will likely employ preconception diagnosis of carrier status to decrease the likelihood of transmitting recessive diseases to their offspring.

At present, carrier genetic screening is limited to patients attending assisted reproduction centers, especially those using donor gametes, where priority is to reduce the risk of hereditary diseases, which can be easily prevented with a timely diagnosis.

Finally, it is essential to remember the ethical limits that must be respected when using genetic carrier screening. Far from having eugenic purposes, this test aims to prevent the transmission of genetic mutations associated with diseases that have a hypothetical negative impact on the individual, the family, and even public health.

## CONCLUSION

Information on the frequency of recessive diseases in Colombia is very scarce; there are only reports of specific diseases in some country populations with high inbreeding rates. The present study identified a high frequency of genetic mutations in the population analyzed by CGT. This screening is a valuable tool that can alert and prevent the transmission of genetic diseases to offspring and reduce treatment costs for the family and public healthcare services. Genetic carrier screening and ART procedures for indicated cases could lead to an evolution in family planning.

It is likely that, in the near future, genetic carrier screening will not be limited to the population undergoing ART but will also be implemented in the population desiring a natural pregnancy. It is important to emphasize that all these possible applications of carrier screening and ART procedures cannot undermine the inherent ethical boundaries. Such boundaries must always prevail in medical practice and scientific work.
